# Editorial: The molecular pathology of cognitive decline: focus on metals

**DOI:** 10.3389/fnagi.2015.00116

**Published:** 2015-06-17

**Authors:** Paul A. Adlard, Roger S. Chung

**Affiliations:** ^1^Synaptic Neurobiology Laboratory, Division of Mental Health, The Florey Institute of Neuroscience and Mental HealthParkville, VIC, Australia; ^2^Biomedical Sciences, Macquarie UniversityMacquarie Park, NSW, Australia

**Keywords:** metals, cognition, Alzheimer's disease, amyotrophic lateral sclerosis, Parkinson's disease, TBI, down syndrome

The series of articles in this special edition reflect much of the current thinking in regards to the role of metals in aging and neurodegeneration, and highlight the deep involvement of metal ions in critical pathways that may contribute to onset/progression of neurological disease. Furthermore, metal homeostasis may impact directly on signaling cascades relevant to cognition, or may indirectly contribute to functional decline via an effect on specific pathologies in the degenerating brain. As such, the behavioral manifestations associated with normal and pathological aging may be remediated by therapeutics that intervene in metal ion dyshomeostasis.

The broader perspective on the role of metal ions in cognition is outlined in the first two articles. Opazo et al. (2014) provide an overview of the role of copper in the CNS, and more specifically review the role of copper on neurotransmission and the ubiquitin proteasome system. These areas find relevance in the apparent sensitivity of the synapse to the “metal milieu” and further, to the potential for an involvement in neurodegenerative disorders. Copper is postulated to regulate the communication between neurons by modifying the protein configuration and strength of neurotransmission within the CNS. The review by Takeda and Tamano (2014) then provides a good overview on the now established role of zinc in synaptic plasticity and cognition. They further explore the impact of the hypothalamic–pituitary–adrenal (HPA) axis on these pathways. In light of the many avenues by which the HPA axis can be activated, then this represents an interesting area for further investigation. Similarly, the precise molecular mechanisms underlying the modulation of zinc signaling across both normal and “pathological” aging remain to be clarified.

The remaining articles are split out according to disease, beginning with ALS. Lovejoy and Guillemin (2014) provide a thorough and compelling overview on the role of metals in ALS, with a specific focus on iron and copper. This review provides a great backdrop for the subsequent papers on ALS, and points toward the potential efficacy of metal-targeting compounds as an avenue for ALS therapy.

Dang et al. (2014) then specifically examine metal levels in the brain and spinal cord of TDP-43 mutant mice, and demonstrated a modulation of metals in association with an impairment in motor function. This is the first report to identify a potential link between TDP-43 and metals, with further study required to elucidate the mechanisms (which are likely to include oxidative stress) underlying the observed phenomena.

Bourassa et al. (2014) tackle the metal: ALS interaction from the perspective of the aggregation of mutant SOD1. After examining cells expressing either WT or mutant SOD1, they discovered a significant cytoplasmic deficit in copper (which was also found in the aggregates themselves) in association with all the mutant forms of the protein. Targeting this metal: SOD1 interaction may thus be a therapeutic approach to limit disease progression. These studies cumulatively add further evidence to the growing literature on the role of metals in ALS.

The next series of articles have a focus on AD, with discussions around both the mechanistic and therapeutic implications of altered metal ion homeostasis. McCord and Aizenman (2014) provide a thorough review on the role of zinc-dependent signaling cascades during normal brain aging and in age-related neurodegeneration, with a particular focus on the production of reactive oxygen species and subsequent oxidative stress. As highlighted in the paper, these are phenomena that are applicable across a broad range of age-related conditions, but which are particularly relevant to AD where Zn has been extensively implicated in the pathogenesis of disease.

Wong et al. (2014) explore the links between metal dyshomeostasis and brain cholesterol in AD, reviewing potential roles in APP processing, Abeta generation/aggregaton/degradation and cell toxicity. They propose a crosstalk between metals and cholesterol in AD pathogenesis. In a related review, Xu et al. (2014) examine the interaction between metals and the major genetic risk factor for AD, ApoE (which is also involved in cholesterol metabolism). The evidence demonstrates that metals bind to ApoE in an isoform-specific way, and that ApoE modulates metal homeostasis in the brain. There is also the possibility that metals may regulate ApoE levels. These data provide support for the interaction between ApoE and metals within the brain, with implications for the pathogenesis of AD. Further exploring a related notion, Flinn et al. (2014) present a research paper examining the interaction of dietary zinc in an AD mouse model, specifically in the context of different ApoE genotypes. The data demonstrate that zinc supplementation caused significant impairments in the AD mouse model containing the ApoE E4 allele. These data are very important in the context of human drug trials and dietary zinc supplementation, as clearly there are genetic confounds (that themselves may interact with or be altered by metals) that may impact on the desired effect and/or disease progressions. This study provides further support for the growing notion that ApoE genotype is a critical factor in clinical trial design.

Hancock et al. (2014) then review the evidence supporting a complex interplay between glia, zinc and synaptic function across “normal” and “pathological” aging. Brewer then presents his inorganic copper hypothesis (Brewer, 2014). This postulates that the ingestion of copper from drinking water and supplement pills, together with a high fat diet, is a primary mechanism to account for the increasing prevalence of AD in the modern world. Furthermore, he notes that zinc deficiency may also be a major contributor to the development of AD, and goes on to review some of his own work in this area of zinc supplementation as a therapeutic avenue in AD (this is a good counter view to that provided by Flinn and colleagues). The hypotheses around the role of metals in AD are many and varied, as are their potential utility as therapeutic targets. This remains an area of active investigation. The next article by Granzotto and Zatta (2014) reviews the potential role of resveratrol as a therapeutic in AD (and potentially normal aging). They focus on how this natural polyphenol may intervene in AD-related pathways, including inflammation, mitochondrial dysfunction and more specifically, those related to oxidative stress and failures in metal ion homeostasis (zinc, iron, copper, and aluminum).

Finally, Bhattacharjee et al. (2014) provide a review on the potential role of aluminum in Alzheimer's disease and go further by demonstrating (through the use of Gene Chip and miRNA array techniques) that an aluminum-enriched diet fostered an upregulation of pro-inflammatory miRNAs in the Tg2576 animal model of AD.

Finishing this section, Braidy et al. (2014) briefly review metal-protein interactions in neurodegenerative diseases and then provide an overview of the various elemental imaging technologies currently available. They then focus on the application of these methods in AD. Ultimately, as spatial resolution and detection sensitivities are improved, we will be afforded greater insight into the role of metals in disease development and progression.

The next three articles focus on the role of metals in other conditions, and utilize a variety of different models. Sun et al. (2013) examined the apoptotic response across age following a traumatic brain injury (TBI), and demonstrated a number of findings. One highlight was a proteomic analysis that identified an injury-dependent increase in Hsp27. This protein, amongst other activities, has been shown to decrease ROS and thus, an endogenous or exogenous increase could protect against metal-mediated oxidative stress and downstream consequences. Another protein, the metal (calcium) binding protein hippocalcin, was also significantly decreased following injury- and this occurred concomitantly with an increase in TUNEL-positive cells in the aged animals. The cumulative impact of apoptosis on neuronal loss post-injury is a likely contributor to the behavioral deficits that accompany TBI, and metals are likely to feature in many aspects/signaling cascades of the TBI brain.

Malakooti et al. (2014) then explore the role of metal ion homeostasis in the cognitive decline present in Down syndrome (DS). Given the overlap between AD and DS, there is good justification to support a metal-mediated aspect to the pathogenesis of DS. Whilst there are obvious candidates, such as APP, that support this link (and the subsequent manifestation of cognitive decline and/or AD), there are many unexplored candidates that are similarly affected by the trisomy on chromosome 21 that deserve further attention (including DSCR1 and ITSN1). The data reviewed demonstrate novel pathways linking DS and metals that may provide novel therapeutic avenues for the disease.

Finally, Chege and McColl (2014) provide a detailed description on the use of *C. elegans* as a model system to explore the underlying mechanisms of PD. They propose that a failure in axonal transport is fundamental, with the subsequent impairment in trafficking of metal ion homeostasis proteins (focusing on iron) resulting in downstream oxidative stress and neuronal loss. They also note the potential for the loss of synaptic connections in this model.

The last manuscript in this special edition is by Marx and Gilon (2014), and presents a different perspective on memory than neuroscientists are typically exposed to. They draw together a discussion around a model that rationalizes the phenomenon of biologic memory in physical-chemical terms and in doing so, invoke a potential role for metal ions.

The sum of these articles, which add to my own recent work in the area, present an overview of the many different pathways through which metals may intersect in the pathogenesis of a variety of neurodegenerative diseases, and which may subsequently manifest (directly or indirectly) in behavioral deficits.

## Conflict of interest statement

The authors declare that the research was conducted in the absence of any commercial or financial relationships that could be construed as a potential conflict of interest.
